# Discovery of proton hill in the phase space during interactions between ions and electromagnetic ion cyclotron waves

**DOI:** 10.1038/s41598-021-92541-0

**Published:** 2021-06-29

**Authors:** Masafumi Shoji, Yoshizumi Miyoshi, Lynn M. Kistler, Kazushi Asamura, Ayako Matsuoka, Yasumasa Kasaba, Shoya Matsuda, Yoshiya Kasahara, Iku Shinohara

**Affiliations:** 1 Institute for Space Earth Environmental Research, Nagoya University, Nagoya, Aichi Japan; 2 Space Science Center, University of New Hampshire, Durham, NH USA; 3 Institute of Space and Astronautical Science, Japan Aerospace Exploration Agency, Sagamihara, Kanagawa Japan; 4 Data Analysis Center for Geomagnetism and Space Magnetism, Graduate School of Science, Kyoto University, Kyoto, Kyoto Japan; 5 Planetary Plasma and Atmospheric Research Center, Tohoku University, Sendai, Miyagi Japan; 6 Graduate School of Natural Science and Technology, Kanazawa University, Kanazawa, Ishikawa Japan

**Keywords:** Magnetospheric physics, Magnetospheric physics

## Abstract

A study using Arase data gives the first observational evidence that the frequency drift of electromagnetic ion cyclotron (EMIC) waves is caused by cyclotron trapping. EMIC emissions play an important role in planetary magnetospheres, causing scattering loss of radiation belt relativistic electrons and energetic protons. EMIC waves frequently show nonlinear signatures that include frequency drift and amplitude enhancements. While nonlinear growth theory has suggested that the frequency change is caused by nonlinear resonant currents owing to cyclotron trapping of the particles, observational evidence for this has been elusive. We survey the wave data observed by Arase from March, 2017 to September 2019, and find the best falling tone emission event, one detected on 11th November, 2017, for the wave particle interaction analysis. Here, we show for the first time direct evidence of the formation of a proton hill in phase space indicating cyclotron trapping. The associated resonance currents and the wave growth of a falling tone EMIC wave are observed coincident with the hill, as theoretically predicted.

## Introduction

How kinetic energy is transferred through cyclotron interactions between cyclotron waves and plasma particles is a universal and important problem in collisionless plasma physics. Here we show the discovery of a proton density enhancement, a so-called a proton hill, in gyro-phase space, in conjunction with a falling tone EMIC emission, using in-situ data from the Arase spacecraft. The existence of the proton hill is direct evidence of the nonlinear interactions between ions and frequency chirping cyclotron waves which are also investigated in laboratory plasmas. The nonlinear cyclotron interaction results in the efficient energy transfer in the collisionless plasma which is directly measured by the present study.

In collisionless plasmas, plasma waves with frequency chirping are universal and play an important role in changing the plasma environment over a wide energy range via wave particle interactions through cross-energy coupling^1^. For example, the nonlinear whistler mode chorus emissions that have frequency drifts with so-called rising or falling tones^2^ in the Earth's Van Allen radiation belts are responsible for the relativistic electron acceleration and loss. EMIC waves with rising and falling frequencies are often found in spacecraft observations in the Earth's magnetosphere^3–5^ as well as in planetary magnetospheres^6–8^ and in the solar wind^9^. The electromagnetic ion cyclotron (EMIC) wave is generated through cyclotron resonance with temperature anisotropic protons. These EMIC waves contribute to the loss process of the energetic protons with a few 10 keV and of the relativistic electrons^10,11^ in the Earth's inner magnetosphere through pitch angle scattering.

The frequency chirping emissions are thought to be generated through a nonlinear resonance with the energetic protons, resulting in efficient pitch angle scattering of the plasma^11^. Nonlinear wave growth theory^12,13^ suggests that the frequency change of the cyclotron waves is caused by the nonlinear resonant currents formed by the cyclotron trapping of the particles. The nonlinear wave growth of the rising and falling tone emissions has been reproduced via self-consistent hybrid simulations^14^, and the spatial and temporal evolution of the nonlinear resonant currents have been discussed. A simulation study on the EMIC falling tone emission has also been performed^15^. The phase trapping of the protons considered in the nonlinear wave particle interactions causes a density depression or enhancement in gyro-phase space, the so-called electromagnetic proton hole or hill, respectively. These proton holes and hills result in the formation of nonlinear resonant currents in the wave's electromagnetic field directions. According to the nonlinear wave growth theory, these resonant currents in the wave electric field direction *J*_*E*_ and in the wave magnetic field direction *J*_*B*_ cause the significant wave growth and frequency drift, respectively, as described in the following equations.

1 $$\frac{\partial {B}_{w}}{\partial t}+{V}_{g}\frac{\partial {B}_{w}}{\partial h}=-\frac{{\mu }_{0}{V}_{g}}{2}{J}_{E}$$

$$c^{2} k^{2} - \omega \left( {\sum\limits_{s} {\frac{{\omega _{{ps}}^{2} }}{{\Omega _{s} - \omega }}} - \frac{{\omega _{{pe}}^{2} }}{{\Omega _{e} }}} \right) = - \mu _{0} c^{2} k\frac{{J_{B} }}{{B_{w} }}$$

where *B*_*w*_, *ω*, *k*, ${V}_{g}=\partial \omega /\partial k$, *t*, *h*, *ω*_*ps*_, ${\Omega }_{s}={q}_{s}/{m}_{s}{B}_{0}$, *μ*_0_, and *c* indicate the wave magnetic field amplitude, wave angular frequency, wavenumber, group velocity, time, distance from equator, plasma frequency of plasma species *s* (*e* represents electron), cyclotron frequency of species *s*, magnetic permeability in vacuum, and the speed of light in vacuum, respectively. These equations represent the wave equation with an external source and the cold dispersion relation modulated by the resonant current. According to these equations, the wave propagating with *V*_*g*_ grows with negative *J*_*E*_ and the wave frequency is modulated by *J*_*B*_^13^. For a wave propagating toward the north (*k* > *0*), the frequency increases with positive *J*_*B*_ and vice versa.

**Figure 1:**
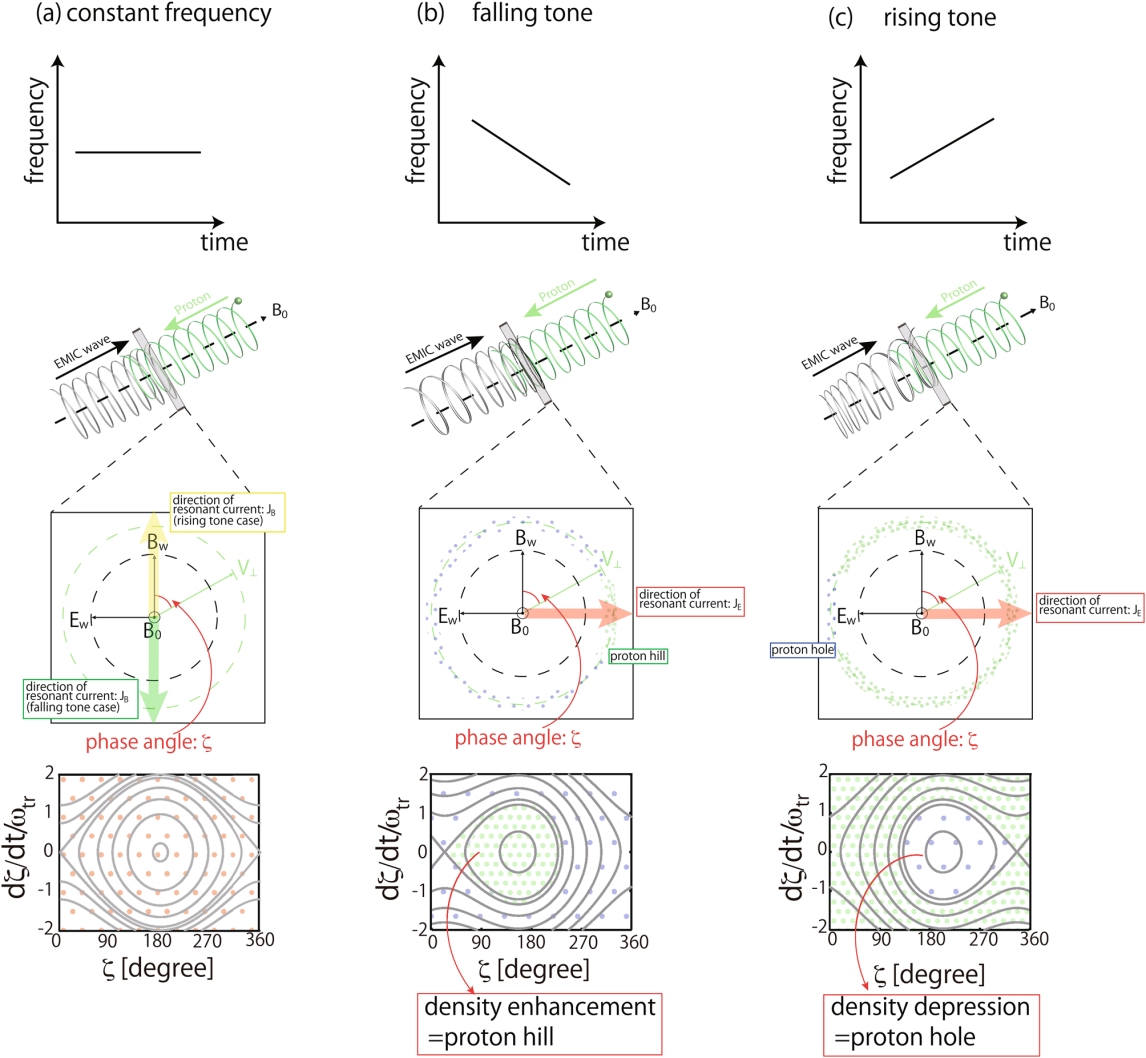
Schematic illustrations of the nonlinear cyclotron interactions of protons with the EMIC waves that have (**a**) constant (*S* = 0), (**b**) falling (*S* < 0), and (**c**) rising (*S* > 0) frequencies. (Created by using Adobe Illustrator 2021, https://www.adobe.com/products/illustrator.html).

According to nonlinear theory, the formation of the nonlinear resonant currents is controlled by the particle motion in phase space^13^. Figure 1 shows a schematic illustration of the phase trapping of the protons with the EMIC waves. Each column of Fig. 1 shows the case with (a) constant frequency, (b) falling tone, and (c) rising tone waves. As shown in the panels in the middle row of Fig. 1, the phase angle *ζ* is defined as the angle between the wave magnetic field *B*_*w*_ and the proton perpendicular velocity *V*_⊥_. The equation of motion of the protons in phase space is as follows^12^.

$$\frac{{d^{2} \zeta }}{{dt^{2} }} = \omega _{{{\text{tr}}}}^{2} \left( {\sin \zeta + S} \right)$$

and

$$\frac{d\zeta }{dt}=-k\left({v}_{\parallel }-{V}_{R}\right)$$

where ${\omega }_{tr}=\sqrt{{kV}_{\perp }{q}_{H}{B}_{w}/{m}_{H}},{V}_{\perp },S$, and ${V}_{R}=(\omega -{\Omega }_{H})/k$ are the trapping frequency, average perpendicular velocity of the protons, inhomogeneity factor and resonance velocity, respectively. The inhomogeneity factor *S* is a function of the frequency sweep rate $\partial \omega /\partial t$ and ambient magnetic field gradient $\partial \Omega _{H} /\partial x$, where *x* is the distance along the ambient magnetic field from the equator. At the equator where the EMIC waves are thought to be generated, the sign of *S* is the same as the frequency sweep rate.

From a pendulum equation modified with the *S* value, the particle trajectories in phase space calculated from Eq. (1), as shown in the lower panels of Fig. 1, are separated into trapped and untrapped regions. Differences in the particle densities between these regions appear because the particles cannot cross the separatrix, and this forms the nonlinear resonant currents. The density enhancement and depression in phase space are called the electromagnetic proton hill and hole, respectively. The shape of the separatrix and resulting resonant current direction are functions of the inhomogeneity parameter *S*. When *S* = *0*, the proton hole or hill appears around $\zeta ={180}^{\circ }$, and then *J*_*B*_ appears in the parallel ($\zeta ={0}^{\circ }$) or anti-parallel ($\zeta ={180}^{\circ }$) direction, respectively. At the equator, the proton hill and hole move when the frequency of the wave drifts, and then the direction of the nonlinear resonant current is changed. Schematic illustrations of the proton hills and holes with negative and positive *S* are shown in Fig. 1b,c. The resultant resonant current *J*_*E*_ direction is also indicated in each middle panel. The proton hill moves toward the smaller *ζ*, while the proton hole moves toward the larger* ζ*. Therefore, even though the frequency rises or decreases, the negative *J*_*E*_ is formed by the inhomogeneity of the proton distribution.

**Figure 2:**
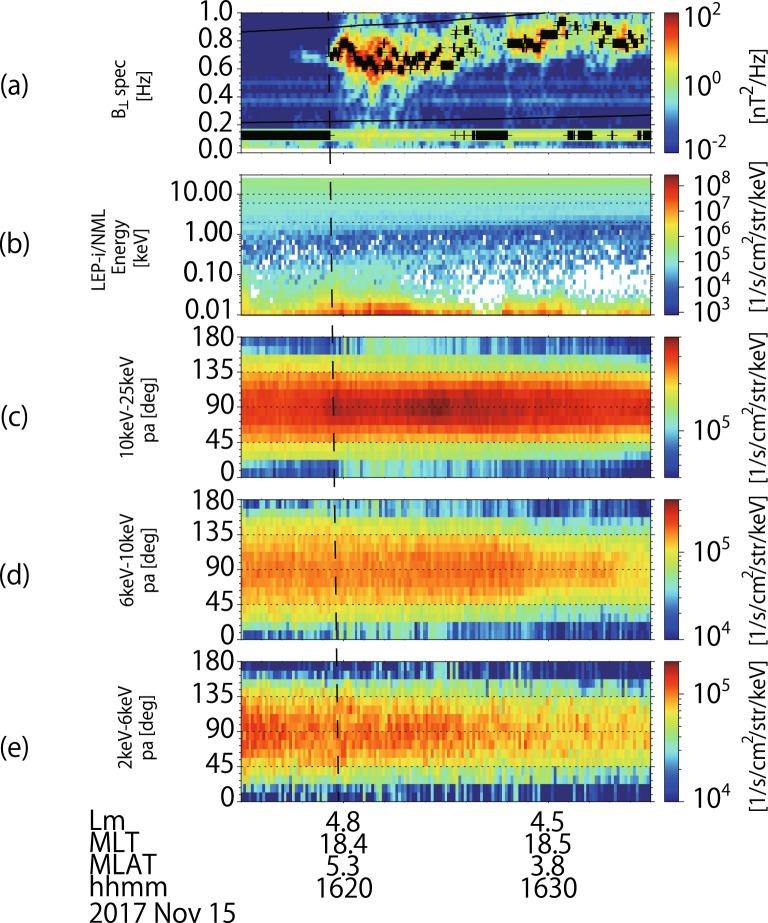
(**a**) Dynamic spectrum of magnetic field in the perpendicular direction to the ambient magnetic field. The black solid lines indicate the helium (upper) and oxygen (lower) cyclotron frequency. (**b**) Energy-time diagram of the proton flux observed by LEP-i. The horizontal dotted lines indicate the energy at 2 keV, 6 keV, 10 keV, and 25 keV, respectively. (**c**–**e**) Pitch angle distributions of the proton flux with energy range 10–25 keV, 6–10 keV, and 2–6 keV, respectively. The satellite positions are described in the bottom of the figures; Lm is McIlwain L-shell of the field line, MLT is the magnetic local time, MLAT is the magnetic latitudes from the magnetic equator along the field line, and hhmm shows the universal time on Nov. 15, 2017. (Created by using IDL 8.8 https://www.l3harrisgeospatial.com/Software-Technology/IDL).

We have surveyed the wave data observed by Arase from March, 2017 to September 2019, and find the best falling tone emission event. On November 15, 2017, the Arase spacecraft observed an EMIC emission around 16:17–16:30. Figure 2a shows the dynamic spectrum of the magnetic field *B*_⊥_ perpendicular to the ambient magnetic field. The dotted line shows the frequency at which the power spectrum density reaches local maximum. The wave emission is just below the helium gyro frequency (upper solid line). During the time interval from 16:17 to 16:22, the wavenormal and Poynting flux analyses, derived from the SVD method^16^, suggest that the wave propagates along the magnetic field mostly to the north from the magnetic equator with an averaged wave normal angle of ~ 3.8°. The polarization (− 0.12) shows L-mode emission indicating that the observed wave is an EMIC wave. In Fig. 2a, we note a monochromatic and weak wave starting around 16:17. From around 16:19 (shown by the vertical dashed line), a complicated frequency change with both frequency increases and decreases is observed. The energy-time diagram in Fig. 2b shows the existence of energetic protons greater than 1 keV which is a possible source population for the observed EMIC wave. We separate the proton flux data into different energy ranges and show the pitch angle distribution in each energy range in Fig. 2c–e. After the wave emission (shown by the vertical dashed line in Fig. 2), the proton flux in the low pitch angle ranges ($<\sim 30^\circ$ and $>\sim 150^\circ$) becomes larger in the higher energy ranges, as shown in Fig. 2c,d.

**Figure 3:**
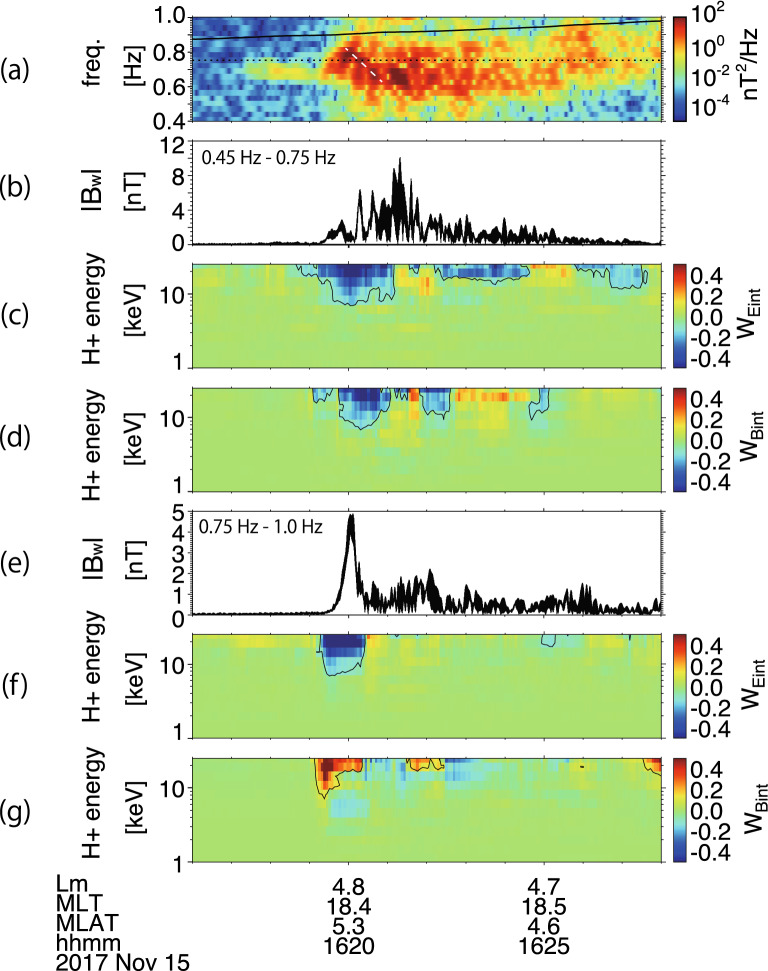
(**a**) Dynamic spectrum of the magnetic field in the spin axis direction. The black line indicates the helium cyclotron frequency. The white dashed line shows the estimated frequency sweep rate. (**b**, **c**, **d**) Magnetic field wave amplitude, ${W}_{Eint}$, and ${W}_{Bint}$ calculated with the electromagnetic field bandpass filtered between 0.45 and 0.75 Hz (shown by dotted line), respectively. Black curves show the contour with the values = − 0.1. (**e**–**g**) Magnetic field wave amplitude, ${W}_{Eint}$, and ${W}_{Bint}$ calculated with the electromagnetic field bandpass filtered between 0.75 and 1.0 Hz, respectively. Black curves show the contour with the values ${W}_{Eint}=-0.1$, and ${W}_{Bint}=0.1$. The WPIA results, ${W}_{Eint}$, and ${W}_{Bint}$, have been calculated with protons of pitch angle between 125° and 145° (Created by using IDL 8.8 https://www.l3harrisgeospatial.com/Software-Technology/IDL).

In the linear theory, the northward propagating EMIC waves can resonate with the counter-streaming protons. Through a parametric survey of the WPIA, we select the pitch angle $\alpha$ range of the protons of $125^{^\circ } \le \upalpha \le 145^{^\circ }$. Figure 3a shows the dynamic spectrum of the magnetic field along the spin axis. The wave emission consists of rising and falling tones, and they appear in the higher and lower frequencies, respectively. To show clear features of the falling and rising tones, we separated the wave into two frequency ranges, 0.45 Hz < *f* < 0.75 Hz and 0.75 Hz < *f* < 1.0 Hz, by a bandpass filter. The WPIA results with the two frequency ranges are shown in Fig. 3b–d and e–g. The three panels of the WPIA results show the magnetic wave amplitude, *W*_Eint_, and *W*_Bint_, respectively. Here, *W*_Eint_ and *W*_Bint_ are $\boldsymbol{V}\cdot \boldsymbol{E}$ and $\boldsymbol{V}\cdot \boldsymbol{B}$ averaged within $\pm 30$ s of each time index. The indices *W*_Eint_, and *W*_Bint_ correspond to the nonlinear resonant currents in the electric and magnetic fields. Since it is difficult to discuss the moment values (density and currents) observed by LEP-i quantitatively because LEP-i measures a part of the distribution function, the WPIA results are normalized by the peak absolute values and are shown in an energy-time diagram. Figure 3b–d are the results for the lower frequency range. The wave amplitude increases with negative *W*_Eint_, and negative *W*_Bint_. Thus, the WPIA result suggests that the falling tone emission occurs with the nonlinear resonant current (${J}_{B}<0$ and ${J}_{E}<0$). Meanwhile, in the higher frequency range, we see negative *W*_Eint_ with positive *W*_Bint_, corresponding to the rising tone wave growth (${J}_{B}>0$ and ${J}_{E}<0$). During the time when significant wave emissions shown in Fig. 3e take place (16:19–16:22) the value of *W*_Eint_ becomes smaller than − 0.1; this is indicated by the contour in Fig. 3c,f, in the energy range > 7 keV. Positive *W*_Bint_ in Fig. 3g appears earlier than the negative *W*_Bint_ in Fig. 3d, indicating that the EMIC emission starts with the rising frequency, which is consistent with the observations. However, after 16:22, the WPIA analyses show different results than expected from the nonlinear theoretical prediction. The estimated electric field in the spin axis becomes larger than the observed electric field in the spin plane after 16:22, and then the observed data after the time is not best for the WPIA.

**Figure 4:**
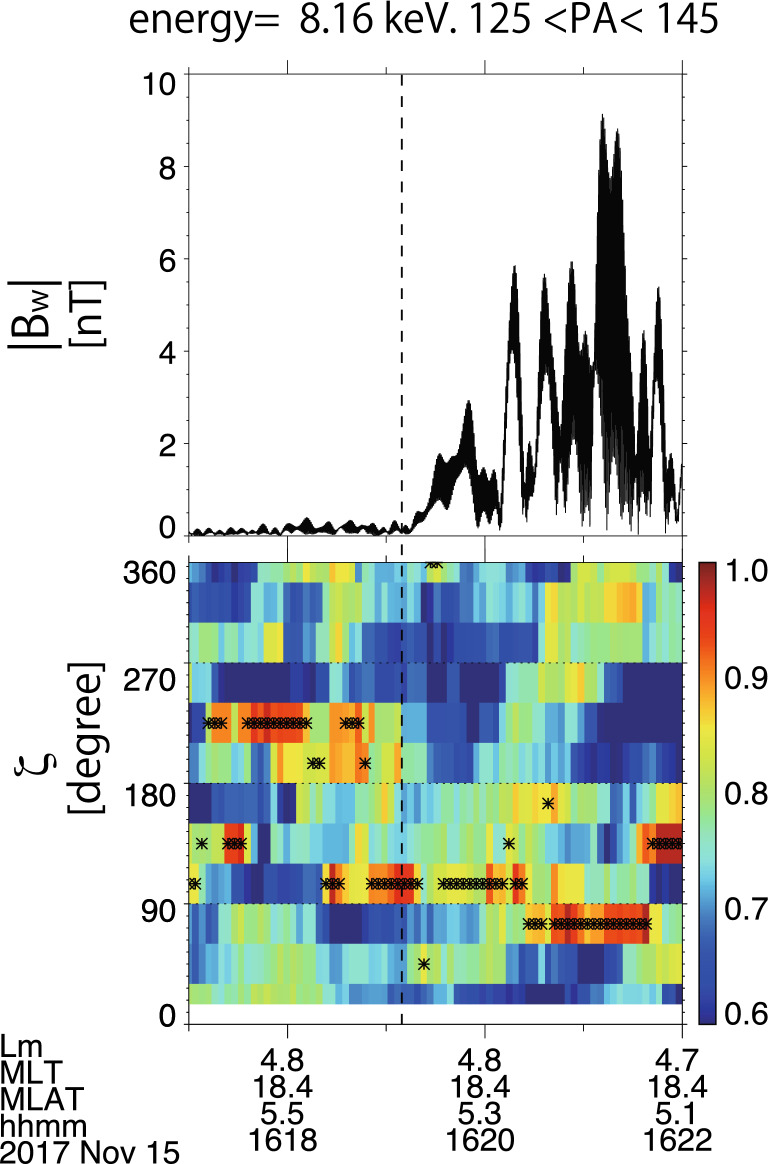
(**a**) The magnetic field wave amplitude between 0.6 and 0.75 Hz. (**b**) Flux distribution of the protons in ζ direction normalized by the maximum value. Dots in the (**b**) indicates the maximum flux in each time. (Created by using IDL 8.8 https://www.l3harrisgeospatial.com/Software-Technology/IDL).

We plot the wave magnetic field amplitude and the proton flux distribution at 8.16 keV in the *ζ* direction in Fig. 4. The dots in the panel (b) indicate the maximum flux at each time. To emphasize the clear interaction with the protons, we limit the wave frequency to the range between 0.6 and 0.75 Hz. In the theory^15^, the nonlinear resonant currents for the falling tone emission are formed by the particle density enhancement in phase space. Initially, the flux enhanced region shown by the dots in Fig. 4b appears around $\zeta =220^\circ$ as shown in Fig. 1a in the early stage of the wave growth. When the nonlinear growth starts, the frequency decreases ($\partial \omega /\partial t<0$) and *S* becomes negative. The enhancement moves toward the smaller $\zeta$ value ($\zeta <180^\circ$). The tendency of the motion agrees with the theoretical change of the proton hill from symmetric (Fig. 1a) to asymmetric (Fig. 1b). In the transition stage from the symmetric hill to the asymmetric hill at around the dashed line in Fig. 4, the angle $\zeta$ of the maximum flux changes to around $90^\circ$ and the wave grows significantly (after the dashed line in Fig. 4). The averaged statistical error is around 0.1, and so the enhancement is significant. The 1-min integration of the flux is too long to capture the fast variations of the proton hill. The significant wave growth can be caused by the nonlinear resonant current ${J}_{E}$ as shown in Eq. (1), which is captured as negative ${W}_{Eint}$ values by WPIA and results in the asymmetric proton hill. Since we select the frequency range in which the falling tone is dominant (0.6 Hz < *f* < 0.75 Hz), the expected proton hole for the higher-frequency rising tone emission is unclear in Fig. 4b.

The averaged frequency sweep rate is − 0.02 rad/s^2^, as shown by the white dashed line in Fig. 3a. Assuming that the resonance energy is 8.16 keV with a pitch angle of $135^\circ$, the inhomogeneity parameter *S* value changes from − 0.3 to − 0.03 as the wave amplitude grows from 1 to 9 nT. An asymmetric proton hill with a finite *S* value is found in Fig. 4b, and results show good agreement with the theoretical prediction^15^.

In conclusion, we apply the WPIA method to a complicated nonlinear EMIC emission observed by the Arase satellite that shows both rising and falling emissions. The WPIA method successfully identified the resonance currents due to wave particle interactions that generated both the rising and falling emissions. For the first time, we successfully identify the flux enhancement of the phase trapped protons, the proton hill, associated with the falling tone. As predicted by nonlinear growth theory, the trapped particles in a limited energy and pitch angle range form the nonlinear resonant currents resulting in the falling tone wave growth. The rising and falling tone emissions interact with the energetic protons in a similar energy range (> 8 keV) causing the pitch angle scattering as shown in Fig. 2.

## Methods

### Wave particle interaction analysis (WPIA) method

Nonlinear EMIC waves with rising and falling frequencies have been observed by Arase. In the present study, we adopt the Wave Particle Interaction Analysis (WPIA) method^17^ for EMIC waves observed by the Arase satellite. In the WPIA method, the phase angles *ζ* between the wave magnetic field and the perpendicular velocity of the particles are calculated, and then we obtain the inner products $\boldsymbol{v}\cdot \boldsymbol{B}$ and $\boldsymbol{v}\cdot \boldsymbol{E}$, which correspond to the nonlinear resonant currents controlling the wave frequency change *J*_*B*_ and the wave growth *J*_*E*_, respectively. We integrate $q\boldsymbol{v}\cdot \boldsymbol{E}$ in velocity space as follows

$$W=\int \int \int \left(q\boldsymbol{v}\cdot \boldsymbol{E}\right)f\left(\boldsymbol{v}\right){d}^{3}\boldsymbol{v},$$

where $q,f(\boldsymbol{v})$ are the charge and the velocity distribution function of particles. We calculate the average of *W* in an observation time range (${t}_{1},{t}_{2}$) as follows

$$W_{{Eint}} = \int\limits_{{t_{1} }}^{{t_{2} }} {Wdt} \sim - q\sum\limits_{\phi } {\Delta \phi } \sum\limits_{\alpha } {\Delta \alpha } \sum\limits_{K} {(\Delta K/K)} \sum\limits_{i} {\sin ~\zeta _{i} \left( \phi \right)\left[ {\sin ^{2} \alpha C_{i} E_{{wi}} /G_{i} } \right]} ,$$

where $\phi ,\alpha ,K,{C}_{i}$ are the gyro phase angle, the pitch angle, the kinetic energy, and the count of an index of a particle detector $i$. The same method is used for ${W}_{Bint}.$ More detail of the WPIA method for the EMIC wave is described in Ref.^18^.

The Arase satellite carries several instruments for plasma particle, magnetic field, and wave observations. In the present study, we use the following instruments onboard the Arase satellite: the low-energy particle detector (LEP-i)^19^ for the 3D flux distribution function data, the plasma wave experiments (PWE)^20^, electric field detector (EFD)^21^ for 256 Hz sampling electric field waveform in the spin plane, and the magnetic field detector (MGF)^22^ for 256 Hz magnetic field waveform in the spin plane and axis. In the WPIA method, the three dimensional electric field is required, while the Arase satellite observes the two dimensional electric field in the spin plane. We confirm the planarity of the wave derived by the SVD method is greater than 0.8 and then we assume the wave as a plane wave which satisfies $\boldsymbol{E}\cdot \boldsymbol{B}=0$ to obtain the electric field in the spin axis. The LEP-i normally observes a full 3D distribution function of multi-species ions within each spin period (*T*_spin_ ~ 8 s). In the spin plane, there are 16 angular bins, and the observation time difference between adjacent bins is *T*_spin_/16. By using the exact time when individual angular bins are measured, we obtain higher time resolution particle data to calculate the *ζ* angle. When we sort the particle data by *ζ* angle, the unevenness of the observation time appears. Then we take an average of the distribution sorted in the *ζ* angle in time. In the present study, we set the integration time to 60 s to obtain enough ion counts in each *ζ* direction. The counts observed by LEP-i averaged in 60 s during the event are around > 60 in each *ζ* bin. Therefore, the error from counting statistics which is proposed in Ref.^17^ is < 12%.

## Data Availability

Data from Arase used in this study are available from the ERG Science Center^23^ operated by ISAS/JAXA and ISEE/Nagoya University (https://ergsc.isee.nagoya-u.ac.jp/data_info/index.shtml.en). The present study analyzed LEP-i L2 v03_00^24^ data for protons, MGF L2 v03.03 (256 Hz/8 s)^25,26^ for magnetic field data, PWE/EFD L2 v03.00 (256 Hz)^27^ for electric field data.

## Code availability

The software used to read and analyze the data, part of the publicly available SPEDAS software package, is available at http://themis.ssl.berkeley.edu/software.shtml and can be used without any restriction.
